# Associations between Plasma Essential Metals Levels and the Risks of All-Cause Mortality and Cardiovascular Disease Mortality among Individuals with Type 2 Diabetes

**DOI:** 10.3390/nu15051198

**Published:** 2023-02-27

**Authors:** Zhaoyang Li, Ruixin Wang, Tengfei Long, Yali Xu, Huan Guo, Xiaomin Zhang, Meian He

**Affiliations:** Department of Occupational and Environmental Health, State Key Laboratory of Environmental Health for Incubating, School of Public Health, Tongji Medical College, Huazhong University of Science and Technology, Wuhan 430030, China

**Keywords:** essential metals, mortality, type 2 diabetes (T2D), cohort study

## Abstract

Epidemiological evidence regarding the possible link between multiple essential metals levels and all-cause mortality and cardiovascular disease (CVD) mortality among type 2 diabetes (T2D) patients is sparse. Here, we aimed to evaluate the longitudinal associations between 11 essential metals levels in plasma and all-cause mortality and CVD mortality among T2D patients. Our study included 5278 T2D patients from the Dongfeng–Tongji cohort. LASSO penalized regression analysis was used to select the all-cause and CVD mortality-associated metals from 11 essential metals (iron, copper, zinc, selenium, manganese, molybdenum, vanadium, cobalt, chromium, nickel, and tin) measured in plasma. Cox proportional hazard models were used to estimate hazard ratios (HRs) and 95% confidence intervals (CIs). Results: With a median follow-up of 9.8 years, 890 deaths were documented, including 312 deaths of CVD. LASSO regression models and the multiple-metals model revealed that plasma iron and selenium were negatively associated with all-cause mortality (HR: 0.83; 95%CI: 0.70, 0.98; HR: 0.60; 95%CI: 0.46, 0.77), whereas copper was positively associated with all-cause mortality (HR: 1.60; 95%CI: 1.30, 1.97). Only plasma iron has been significantly associated with decreased risk of CVD mortality (HR: 0.61; 95%CI: 0.49, 0.78). The dose-response curves for the association between copper levels and all-cause mortality followed a J shape (*P*_for nonlinear_ = 0.01). Our study highlights the close relationships between essential metals elements (iron, selenium, and copper) and all-cause and CVD mortality among diabetic patients.

## 1. Introduction

Diabetes mellitus (DM), as a major public health worldwide, has shown a steady and rapid growth trend of prevalence during the past few decades. In China, the latest research based on a national data showed that the estimated prevalence of diabetes has significantly increased from 10.9% in 2013 to 12.4% in 2018 [1]. Sustained high levels of blood glucose can cause a series of complications, and type 2 diabetes (T2D) patients have higher mortality risk than the general population [2]. As one of the top ten causes of death globally [3], diabetes directly caused 1.5 million deaths and 48% of all deaths due to diabetes occurred before the age of 70 years in 2019 [4]. Therefore, it is urgent to explore the factors related to the mortality in diabetic patients from a comprehensive perspective.

Essential trace elements, such as iron, zinc, and copper, are required by living organisms, including human beings [5]. However, only appropriate levels of these elements can maintain the best functional state of health [6]. More and more research is linking essential trace elements and all-cause mortality and CVD mortality in general population. For example, Shi et al. [7] found significantly positive associations between copper, molybdenum, and vanadium and all-cause mortality and CVD mortality and negative associations between selenium and all-cause mortality and CVD mortality among the general Chinese population. In a 5-year follow up study, Long et al. [8] found inverse associations between zinc and selenium and incident CVD risk in patients with T2D. However, evidence for the association between essential trace elements levels and mortality among individuals with T2D are limited. As far as we are aware, only one recent study using 2003–2004 and 2011–2014 data from the National Health and Nutrition Examination Survey (NHANES) examined the association of serum selenium concentrations with all-cause mortality and heart disease mortality among individuals with T2D and reported a negative association between them [9]. However, humans are exposed to a variety of essential metal elements simultaneously in real life. It is indicated that the levels of essential metals, such as zinc and copper, are different between those with diabetes and healthy individuals [10]. Accordingly, more investigations are needed to further illustrate potential associations between multiple essential trace elements and all-cause mortality and CVD mortality among T2D patients.

Therefore, in the current study, we aimed to conduct a cohort study to assess the potential associations between 11 essential metals and the incidence of all-cause mortality and CVD mortality among T2D patients derived from the Dongfeng–Tongji cohort.

## 2. Materials and Methods

### 2.1. Study Subjects

All study participants were from the Dongfeng–Tongji (DFTJ) cohort, which has been described previously [11]. The DFTJ cohort is a prospective cohort study initiated between September 2008 and June 2010 with the enrollment of 27,009 retired workers of the Dongfeng Motor Corporation. Information on lifestyle, medical history, and health conditions was updated every 5 years by means of questionnaire and physical examination. For the current analysis, subjects diagnosed with type 2 diabetes at baseline (*n* = 5173) and during the first follow-up period (*n* = 1509) were enrolled initially. Participants were excluded if they had insufficient blood samples (*n* = 404), CHD and stroke at baseline (*n* = 401), cancer at baseline (*n* = 190), missing information on BMI, smoking status, drinking status, education information (*n* = 129), fasting blood glucose (FBG) level (*n* = 243), duration of diabetes (*n* = 22), estimated glomerular filtration rate (eGFR) level (*n* = 14), and were lost to follow up (*n* = 1). Finally, 5278 subjects were included in the present study (Figure S1).

The present study was approved by the Medical Ethics Committee at the School of Public Health, Tongji Medical College, Huazhong University of Science and Technology in 2008 (approval no: 2008-03). All participants gave their written informed consent.

### 2.2. Measurement of Metals Exposure

Plasma levels of the 11 essential metals, including zinc, selenium, manganese, molybdenum, vanadium, cobalt, chromium, nickel, and tin, were measured by the quadrupole inductively coupled plasma-mass spectrometry (ICP-MS, Agilent 7700 × series; Agilent Technologies). Details of the procedure and the limits of detection (LOD) have been described by Long et al. [8], and the percentages of samples below detection limits of study participants at baseline in the current study are displayed in Table S1. In the samples with metal levels below the detection limit, we imputed the metal levels using half of the detection limit.

### 2.3. Assessment of Covariates

Data on age, gender, lifestyle (smoking status, alcohol drinking status, and physical activity), presence of hypertension, hyperlipidemia, CVD, cancer, medical history (such as antihypertensive drugs and lipid-lowering medication), and family history of disease were obtained by a face-to-face interview based on demographic questionnaire. Body mass index (BMI) was calculated as weight in kilograms divided by the square of height in meters (kg/m^2^), which were measured during the process of physical examination. More detailed information has been described previously [11].

The level of FBG, serum lipids (including triglyceride (TG), total cholesterol (TC), high-density lipoprotein (HDL), low-density lipoprotein (LDL), and the blood pressure value (including systolic blood pressure (SBP) and diastolic blood pressure (DBP)) were measured at baseline. In addition, eGFR were calculated according to the Modification of Diet in Renal Disease equation [12].

### 2.4. Ascertainment of Type 2 Diabetes

T2D was ascertained according to the American Diabetes Association (ADA) criteria [13] if the participants meeting any of the following criteria: (1) self-reported physician’s diagnosis of diabetes in community hospitals or higher-level hospitals; (2) FBG level ≥ 7.0 mmol/L; (3) HbA1c level ≥ 6.5%; and (4) usage of diabetes medication (insulin or oral hypoglycemic agent).

### 2.5. Ascertainment of All-Cause and CVD Mortality

The confirmation method of mortality status has been described previously [7]. Briefly, all-cause deaths and CVD deaths were obtained by integrating the information recorded in the medical insurance system of Dongfeng Motor Company with the questionnaire and survival physical examination information during the follow-up. Meanwhile, the specific causes of death were ascertained and classified following the International Classification of Diseases Tenth Revision (ICD-10) by the trained staff who were blinded to the participant questionnaire data.

### 2.6. Statistical Analyses

Differences in the basic characteristics between deaths and survivors were analyzed by using the Student’s *t*-test or Mann–Whitney U test for continuous variables, and Chi-squared test for categorical variables. The correlations between essential metals were assessed by using the Spearman’s rank correlation analyses. Concentrations of the plasma essential metals were natural logarithm (ln), due to their skewed distributions.

Considering the correlations among the plasma metals, the least absolute shrinkage and selection operator (LASSO) penalized regression analyses were first used to select the most significant metals associated with all-cause mortality or CVD mortality. Then, the significant metals derived from LASSO model were included in the Cox proportional hazards models to estimate hazard ratios (HRs) and 95% confidence intervals (CIs) for the associations of metals with all-cause mortality or CVD mortality. We adjusted for the following variables based on a priori knowledge of their potential associations with exposure and outcome: age (years), gender (male/female), BMI, education (primary or below/junior high school/high school/college or above), smoking status (current smoker/ex-smoker/never smoker), drinking status (current drinker, ex-drinker and never drinker), physical activity status, presence of hypertension, presence of hyperlipidemia, baseline FBG, duration of diabetes, and use of antidiabetics and eGFR at baseline. The family history of CVD was additionally adjusted on the basis of the above covariates when the outcome was CVD mortality.

Restricted cubic spline regression (RCS) linked to Cox regression models were conducted to explore the potential non-linear relations between continuous plasma metals concentrations and all-cause mortality or CVD mortality. Knots were set at the 20th, 40th, 60th and 80th percentiles in the models, and the reference value was set to the 10th percentile.

Subjects were further stratified by age (<65 or ≥65 years), gender (women or men), BMI (<24.0 or ≥24.0 kg/m^2^), smoking status (current smoker, ex-smoker and never smoker), drinking status (current drinker, ex-drinker and never drinker), eGFR levels (<90 mL/min/1.73 m^2^ or ≥90 mL/min/1.73 m^2^), diabetes duration (<5 or ≥5 years), presence of hypertension, and presence of hyperlipidemia. The *p* values for the product terms between continuous metals levels, and the stratification variables were used to estimate the significance of interactions.

In addition, we estimated the combined associations between metals that were significantly associated with incident all-cause mortality in the multiple-metals model, and the metal level was dichotomized as low (Quartile1 + Quartile2) and high (Quartile3 + Quartile4), and a four-category variable was created for two metals (i.e., low/low, high/low, low/high, and high/high), with the lowest risk group as the reference.

Sensitivity analyses were conducted to test the robustness of our results. First, we excluded subjects who died within the first 2 years of follow-up to reduce the potentially reverse causation. Second, we excluded subjects with eGFR levels lower than 60 mL/min/1.73 m^2^ at baseline. Third, we excluded the accidental death during the follow-up.

All data were analyzed using SPSS software (version 22.0, SPSS Inc., Chicago, IL, USA) and R software (version 3.6.1, R Foundation for Statistical Computing, Vienna, Austria). LASSO penalized regression analyses were performed using the “glmnet” and “survival” package in R. A two-sided *p*-value < 0.05 was considered statistically significant.

## 3. Results

### 3.1. Characteristics of Study Participants

During 44,017.87 person-years of follow-up, a total of 890 deaths (including 312 CVD deaths) were identified. Compared with survivors, those who died during the follow-up period were more likely to be older, male, with a higher percentage of hypertension and hyperlipemia and longer duration of diabetes. Meanwhile, those who died during the follow-up period were more likely to be current smokers, current drinkers, and less physically active; in addition, their levels of FBG were higher, but eGFR were lower, in contrast to those survivors (Table 1).

Participants who died during the follow-up tended to have lower levels of zinc and selenium, but higher levels of manganese, molybdenum, vanadium, cobalt, nickel, and tin (all *p* values < 0.05). The correlations between the 11 metals were indicated in Figure S2. Specifically, most of the metals were significantly correlated with each other, except for selenium and nickel, selenium and vanadium, selenium and manganese, iron and molybdenum, and zinc and copper (all *p* values > 0.05). The absolute value range of the correlation coefficients between metals is 0.1–0.68.

### 3.2. Association between Plasma Metals Levels and All-Cause Mortality

According to the results from LASSO models (Table S2), five metals, including iron, copper, zinc, selenium, and molybdenum, were the most significant metals associated with all-cause mortality. Among them, the highest coefficient is allocated to selenium (coefficient = −0.399), followed by copper (coefficient = 0.358), iron (coefficient = −0.123), zinc (coefficient = −0.020), and molybdenum (coefficient = 0.023), respectively.

We further conducted multiple-metals models by including the above five metals simultaneously in the models (Table 2). Ln-transformed metals were included as continuous variables in the model, and we examined the associations between one unit increase of Ln-transformed metals levels and all-cause mortality. The corresponding adjusted HRs (95%CIs) were 0.83 (0.70,0.98), 1.60 (1.30,1.97), and 0.60 (0.46,0.77) for iron, copper, and selenium, respectively. Meanwhile, compared with the lowest quartile of plasma metals, the multivariable-adjusted HRs (95%CIs) of all-cause mortality in the highest quartile were 0.79 (0.64,0.96), 1.50 (1.23,1.82), and 0.72 (0.58,0.88) for iron, copper, and selenium, respectively. However, no significant association between zinc and molybdenum and all-cause mortality were observed.

**Figure 1:**
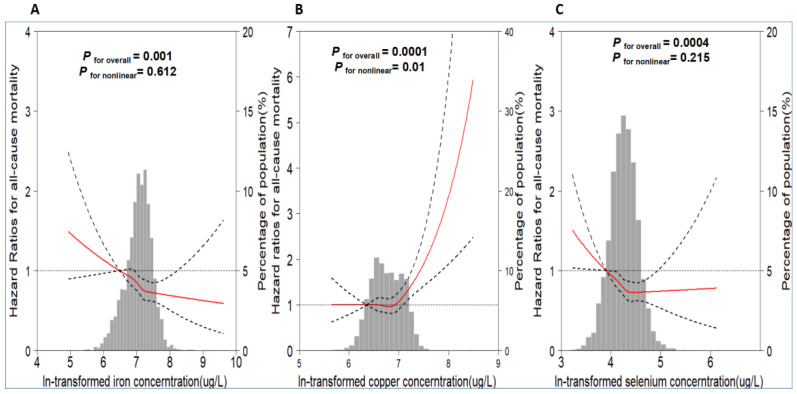
The restricted cubic spline for the association between plasma metals ((**A**): iron; (**B**): copper; (**C**): selenium) and all-cause mortality among type 2 diabetes individuals. Knots were placed at the 20th, 40th, 60th and 80th percentile of the distribution of plasma metal concentration, with the reference value were set at the 10th percentile. The black line was hazard ratio; dashed lines were 95% confidence intervals. Adjustment factors were age, gender, BMI, education, smoking status, drinking status, physical activity status, baseline FBG, duration of diabetes, and use of antidiabetics and eGFR at baseline.

Further cubic splines analysis revealed a significant nonlinear dose–response relation (J shaped) between copper and all-cause mortality (*P*_for nonlinearity_ = 0.01, Figure 1B), and a surge was observed when the levels of copper are around 1006.88 μg/L. Significant linear associations between iron (*P*_for overall_ = 0.001 with *P*_for nonlinearity_ = 0.612) and selenium (*P*_for overall_ = 0.0004 with *P*_for nonlinearity_ = 0.215; Figure 1A,C) and all-cause mortality were observed.

Subgroup analyses (Figures S3–S5) indicated that the positive association between copper levels and all-cause mortality was more pronounced among subjects with lower levels of BMI and eGFR and with shorter duration of diabetes (*P*_for interaction_ = 0.015, 0.002 and 0.017, respectively; Figure S4), and the negative association between selenium levels and all-cause mortality was more pronounced among subjects who were younger and with lower levels of eGFR (*P*_for interaction_ = 0.003 and 0.024, respectively; Figure S5). No significant interactions were found for iron and the stratified factors.

In addition, sensitivity analyses indicated that association between plasma selenium, copper and all-cause mortality remained largely unchanged when we excluded participants who died within first 2 years of follow-up or participants with eGFR levels lower than 60 mL/min/1.73 m^2^ or who died accidentally during follow-up, although the inverse associations between iron and all-cause mortality did not reach statistical significance because of decreased power caused by the reduction of the sample size (Table S3).

Metals interaction analyses did not observe any significant interaction between plasma copper, selenium, and iron (all *P*_for interaction_ > 0.05). However, in the joint association analysis of multiple metals, we did observe that individuals with high levels of iron and selenium had a significantly lower risk of all-cause mortality than those with low levels of iron and selenium (HR = 0.66, 95%CI: 0.55, 0.79; *p* value < 0.001; Table 3).

### 3.3. Association between Plasma Metals and CVD Mortality

Iron was the only significant metal associated with CVD mortality from the LASSO models; the coefficient of iron was −0.306 (Table S2). The Cox regression models revealed that each one unit increase in ln-transformed plasma iron (HR: 0.61; 95%CI: 0.49,0.78) were significantly associated with decreased CVD mortality (Table 3). The multivariate-adjusted HRs (95%CIs) across quartiles of plasma iron concentrations were 1.00 (reference), 0.70 (0.51,0.95), 0.69 (0.51,0.93), and 0.52 (0.38,0.72) for CVD mortality (*P*_trend_ < 0.001). No significant non-linear associations for iron were observed (*P*_for overall_ = 0.0005 with *P*_for nonlinearity_ = 0.914) from the restrict cubic spline analysis (Figure S7). Subgroup analyses (Figure S6) indicated that the negative association between iron levels and CVD mortality were more pronounced among subjects with higher levels of BMI and subjects without hypertension (*P*_for interaction_ = 0.01 and 0.003, respectively). The sensitive analyses showed that the above negative associations were essentially unchanged when we excluded participants who died within the first 2 years of follow-up or participants with eGFR levels lower than 60 mL/min/1.73 m^2^ or who died accidentally during follow-up.

## 4. Discussion

In this study of 5278 T2D individuals with a median follow up of 9.8 years, we found plasma copper was associated with increased risk of all-cause mortality, while plasma selenium was associated with decreased risk of all-cause mortality. Meanwhile, we found that plasma iron was significantly and negatively correlated with both all-cause and CVD mortality. In addition, the dose–response curves for the association between copper levels and all-cause mortality followed a J shape. Up to our knowledge, this was the first investigation to evaluate the prospective associations between plasma multiple essential metals and all-cause and CVD mortality among individuals with T2D.

### 4.1. Copper

Copper, as the third most abundant essential metal in the human body after zinc and iron, is involved in many physiological pathways and plays important roles in physiological processes [14]. Nonetheless, it is also crucial to remark that both copper deficiency and overload play key roles in the occurrence and development of many diseases. For example, copper deficiency is closely related to diseases such as Menkes disease and non-alcoholic fatty liver disease [15], while copper overload can also be closely related to cardiovascular diseases and cancer [16]. Accumulating evidence has supported the positive association between copper levels and all-cause mortality in the general population. For example, the most recent cohort study based on the general population in China reported positive associations between plasma copper and all-cause mortality [7]. Similar conclusions were also derived from early studies: Bates et al. [17] and Mursu et al. [18] reported positive associations between dietary copper intake and all-cause mortality. In addition, two prospective studies also reported significant association between higher levels of serum copper concentrations and all-cause mortality [19,20]. All the above conclusions are similar to those of diabetes patients in this study. The positive correlation between copper and all-cause mortality is reasonable. High levels of free copper are related to excessive oxidative stress [21]. The main copper binding protein in plasma is ceruloplasmin. An in vitro study has shown that chronic hyperglycemia can damage the copper-binding properties of ceruloplasmin [22], which may increase the level of plasma-free copper, resulting in excessive oxidative stress and a series of subsequent adverse health effects. In vivo and in vitro experiments also revealed that copper exposure can promote intimal thickening caused by vascular injury, promote LDL uptake in macrophages and finally promote the occurrence of atherosclerosis [23]. Moreover, metformin, a very common drug used by diabetes patients, had a strong affinity for copper and could play a role in inhibiting blood glucose production through interaction with mitochondrial copper [24].

Notably, we found a significant non-linear relationship of a J shape between copper levels and all-cause mortality among diabetes patients. Specifically, when copper level exceeded about 1006.88 μg/L, the HR significantly increased. This level is within the serum concentration range of the general population (635–1589 μg/L) [25], and the concentration is also slightly lower than the serum copper concentration level (1230 μg/L) in one previous study [20], which also reported a significant positive association between serum copper levels and all-cause death risk in the general population from Germany. This may reflect the increased susceptibility of diabetes patients to environmental exposure compared with the general population to a certain extent. In addition, the study found the positive association between higher copper and increased all-cause mortality risk was more pronounced among subjects with lower levels of BMI and eGFR and with shorter duration of diabetes. Considering the positive association between BMI and diabetes duration and mortality [26,27], the higher levels of BMI and the longer duration may mask the effects of copper on all-cause mortality. Lower levels of eGFR are independently associated with an increased risk of all-cause mortality [28], and elevated circulating copper levels have been associated with decline in kidney function [29]. Therefore, the synergistic interaction between lower eGFR levels and higher plasma copper levels is reasonable.

### 4.2. Iron

Iron, the first most abundant essential metal in the human body, is involved in a wide variety of important cellular processes. However, excessive or lack of iron can be harmful to the body [30]. To our knowledge, although several studies have focused on the iron status and mortality in general population, scarce data are available about iron levels and mortality among diabetic patients [31,32]. One small cohort study among 287 patients with both T2D and coronary artery disease reported a U-shaped relationship between serum ferritin levels and all-cause mortality, but a negative linear association between transferrin saturation (serum iron level/total iron-binding capacity (TIBC) × 100%) and all-cause mortality [33]. One subsequent prospective study among 8003 US adults reported that prediabetes individuals with elevated transferrin saturation had substantially increased mortality risk [34]. In the current study, we found significant inverse associations between plasma iron level and all-cause mortality and CVD mortality. Possible reasons for the inconsistency of the above results may be attributed to differences in exposure range, sample size, and differences in population characteristics, as well as variation in biomarkers selected in different studies. Among these possible factors, it is particularly noteworthy that the selected markers are different. Although iron in plasma only accounts for 0.1% of the total iron in human body, it is of great significance to meet the daily needs of erythropoiesis [35]. Erythropoiesis is crucial to maintain hemoglobin levels. One prospective study based on the general older population indicated that the decline of hemoglobin levels was an independent risk factor for death risk [36]. Thus, the significant association between higher plasma iron and decreased all-cause mortality and CVD mortality found in the present study is reasonable. However, both low iron intake and high iron intake were associated with an increased risk of mortality in Chinese women, derived from one prospective cohort study with a mean follow-up of 9.9 years [37], while dietary intake of total iron was positively associated with mortality from stroke and total CVD in Japanese men, derived from one cohort study (*n* = 58,615) with a median follow-up of 14.7 years [38]. To summarize, more studies using indices, such as serum ferritin, transferrin, and transferrin saturation, which can reflect iron status comprehensively, are needed.

Subgroup analyses indicated that the negative association between iron levels and CVD mortality were more pronounced among subjects with higher levels of BMI and without hypertension. However, epidemiological studies indicated that the serum iron level of adults with higher BMI is lower [39]. Moreover, one recent study showed that compared with diabetes patients with lower BMI (<30 kg/m^2^), the transferrin saturation of subjects with higher BMI (>30 kg/m^2^) was significantly lower, which may suggest the possibility of lower serum iron levels among T2D patients with obesity [40]. We could not explain the potential synergy between higher levels of BMI and plasma iron shown by the interaction analysis. One possible explanation is the negative association between lower BMI and the risk of CVD mortality masked the role of iron in reducing the risk of CVD mortality, and more studies are needed to further illustrate this unexpected finding. Hypertension was reported to be a widely accepted risk factor for all-cause mortality worldwide and is associated with an increased risk of CVD [41], and thus may play an antagonistic role in the process of iron reducing the risk of CVD mortality.

### 4.3. Selenium

Selenium, as another important trace element, was found to play pivotal roles in many physiological processes, including maintenance of normal function of endogenous antioxidant system, thyroid hormone metabolism, and immunological and anti-inflammatory process. Accordingly, the association between selenium status and healthy effects have been widely characterized. Currently, much research links selenium levels to all-cause mortality or cardiovascular mortality, based on observational studies and post hoc analyses of randomized controlled trials among the general population. One recent meta-analysis [42] based on 12 previous observational studies revealed that low selenium level was associated with an increased risk of all-cause mortality risk. Another meta-analysis [43] of 43 randomized controlled trials emphasized that antioxidant mixtures can reduce the risk of all-cause mortality only when selenium was part of the mix. As far as we know, only one study [9] investigated the associations between serum selenium concentrations and the risk of all-cause mortality and heart disease mortality among US adults with T2D. The authors indicated that higher serum selenium concentrations were associated with lower all-cause mortality and heart disease mortality. Our study confirmed the inverse associations between plasma selenium level and all-cause mortality. Meanwhile, a similar linear dose-response relationship was also observed in a relatively narrow concentration range (concentration rang: 50.4–148.6 μg/L in the present study vs. 89–182 μg/L in the above study). Although a considerable variability about the curve was observed when the concentration range of selenium is higher than 148.6 μg/L, this is largely due to the small size of subjects with plasma concentrations greater than 148.6 μg/L (*n* = 39). The potential mechanisms underlying the inverse association between selenium and all-cause mortality have not yet been elucidated in detail. As an antioxidant in the form of selenoprotein, selenium can reduce the production of ROS and inflammation. In vivo studies have also found that supplementation of selenium can not only enhance the activities of superoxide dismutase (SOD), glutathione peroxidase (GSH-Px), but also reduce the levels of pro-inflammatory cytokines, such as IL-1, IL-6, TNF-a, and INF-γ [44]. Additionally, a greater decline in all-cause mortality associated with plasma selenium among younger subjects and individuals with higher level of eGFR were observed. Older people are prone to high risk of insufficient intake of selenium due to loss of appetite and reduced feeding and digestive capacity [45]. Low serum selenium levels were observed in patients with advanced kidney disease in early studies [46], which might explain the negative association between selenium levels and CVD mortality being more pronounced among younger subjects and subjects with higher levels of eGFR.

Although no significant interactions were found, we did observe that subjects with high iron and selenium had a lower risk of all-cause mortality among individuals with T2D. In addition to the close relationship between selenium and iron and the occurrence and development of diabetes [47], these two elements also play an important role in thyroid function [48], bone integrity [49], and many other aspects. Moreover, insulin-transferrin-selenium (ITS), as a basal medium supplement, is sometimes supplemented during routine serum-free bovine cell and embryo culture [50,51], suggesting the significance of iron and selenium in maintaining cell survival. However, more experimental efforts are needed to explore the combined exposure dose and potential mechanisms underlying the potential combined effect between iron and selenium in the future.

### 4.4. Other Essential Elements

In addition to the above three metals, no significant association was found between the other eight metals (manganese, molybdenum, vanadium, cobalt, chromium, nickel, and tin) and all-cause mortality or CVD mortality among individuals with T2D. Although zinc and molybdenum were selected using the LASSO regression, no significant associations between zinc or molybdenum and all-cause and CVD mortality were observed in further multiple-metals model analysis. There is very limited population-based evidence on the relationship between zinc and diabetes death. One previous study found that lower levels of zinc were significantly and positively correlated with the risk of CHD risk in diabetes patients [52]. In vitro experiments showed that zinc supplements could inhibit the vascular calcification of vascular smooth muscle cells exposed to high levels of glucose [53]. The prevalence of vascular calcification is far greater in DM patients than those without DM, and vascular calcification is a well-established independent predictor of CVD [54]. Considering CVD is the number one cause of death in diabetes cases and that, in the present study, the baseline plasma zinc level of the survivors was significantly higher than that of the subjects who died during the follow-up, zinc may have a protective effect on reducing the mortality risk of diabetes patients. In addition, although plasma zinc is a reliable biomarker for reflecting zinc status in healthy people [55], inflammatory status and medication can affect plasma zinc level [56], suggesting that plasma zinc may not be the best biomarker for reflecting zinc status in disease status, including in diabetes. Thus, more prospective studies using appropriate biomarkers are warranted to further explore and confirm. As for molybdenum, no studies on the relationship between molybdenum level and long-term health effects of diabetes have been published to date. Only one recent population study found that high plasma molybdenum levels were significantly and positively correlated with all-cause mortality and CVD deaths in the general population [7]. However, one early study based on the Chinese population found that molybdenum supplementation was negatively correlated with cerebrovascular disease death [57]. In addition, early animal studies also found that molybdate can improve the immune capacity of diabetes rats and restore the ability of antioxidant enzymes in rats [58]. These differences may be attributed to variation in study design and methodology, as well as inherent differences in population characteristics (e.g., exposure levels, disease state and nutritional factors). Thus, further research is warranted to clarify the exact associations between molybdenum levels and all-cause mortality and CVD mortality among diabetes patients.

The current study has several strengths. First, to our knowledge, this is the first prospective study to evaluate associations between 11 essential elements and all-cause mortality and CVD mortality in a middle-aged and older Chinese population with T2D. Second, LASSO models were used to select metals highly associated with all-cause mortality and CVD mortality, which is better than the traditional regression method for the situation of multiple metals with high correlation [59]. In addition, the outcomes were obtained according to strict medical and death records. However, some limitations should also be addressed. First, plasma selenium levels reflect the short-term status rather than the long-term status measured in the whole blood or erythrocyte [60]. However, one previous study [61] derived from the DFTJ cohort reported a significant positive correlation between plasma and whole blood levels of selenium (r = 0.52, *p* < 0.001). Iron, ferritin, transferrin, and transferrin saturation are more suitable to assess the body’s stores than plasma iron level and have been widely used in many studies. Unfortunately, these indices were not measured in the present cohort. Thus, more prospective studies using appropriate biomarkers are warranted to further explore and confirm. Additionally, we only measured the concentration of each metal once at baseline. Nevertheless, most metals including selenium levels showed a good repeatability at baseline (in 2008) and first follow-up (in 2013) in our previous study [61]. Second, we were unable to directly assess the glycemic control and severity of diabetes. However, we have adjusted for the duration of diabetes, baseline blood glucose level, and use of drugs for the treatment of diabetes in the analysis, and the results remained significant. Third, the plasma levels of metals can be influenced by dietary intake. Unfortunately, detailed information about the diet patterns is unavailable in our study.

## 5. Conclusions

Findings from the present perspective cohort study suggest that higher plasma copper levels are associated with a higher risk of all-cause mortality, while higher plasma selenium and iron levels are associated with a lower risk of all-cause mortality among participants with diabetes. In addition, higher plasma levels of iron are also associated with decreased risk of CVD mortality. These data suggest the importance of monitoring the level of copper and supplementation of iron and selenium in the prevention of mortality among individuals with diabetes. Further prospective and experimental studies are warranted to confirm these findings and clarify the underlying mechanisms of these metals on CVD and all-cause mortality among individuals with T2D.

## Figures and Tables

**Table 1:** Participants’ characteristics according to the vital status at the end of the follow-up (*n* = 5278).

	Death (*n* = 890)	Survivors (*n* = 4388)	*p*
Age (years)	69.41 ± 7.15	64.04 ± 7.12	<0.001
Gender (man, %)	61	43	<0.001
BMI (kg/m^2^)	25.30 ± 3.50	25.54 ± 3.36	0.054
Education			<0.001
Primary or below	42.8	30.7	
Junior high school	29.3	38.2	
High school	18.5	21.5	
College or above	9.3	9.4	
Smoking (%)			<0.001
Current smoker	23.8	13.9	
Ex-smoker	19.2	12.4	
Never smoker	57	73.7	
Alcohol consumption (%)			<0.001
Current drinker	18.5	18.3	
Ex-drinker	11.1	6.6	
Never drinker	70.3	75.1	
Physical activity (yes, %)	86.2	89.8	0.002
Hypertension (yes, %)	79.9	70.9	<0.001
Hyperlipidemia (yes, %)	70.4	66.8	0.035
Family history of CVD (yes, %)	6.4	11.3	<0.001
Antidiabetic drugs (yes, %)	50.8	38.5	<0.001
Systolic blood pressure (mmHg)	136.97 ± 20.06	135.89 ± 19.88	0.139
Diastolic blood pressure (mmHg)	77.17 ± 12.29	78.51 ± 11.34	<0.001
Fast blood glucose (mmol/L)	7.70 (6.70,9.68)	7.40 (6.63,8.60)	<0.001
Duration of diabetes (years)	3.27 (0.00,11.23)	0.05 (0.00,7.19)	<0.001
Triglyceride (mmol/L)	1.80 ± 1.73	1.77 ± 1.59	0.61
Total cholesterol (mmol/L)	5.18 ± 1.19	5.17 ± 1.09	0.613
High-density lipoprotein (mmol/L)	1.32 ± 0.38	1.38 ± 0.39	<0.001
Low-density lipoprotein (mmol/L)	3.01 ± 0.95	2.99 ± 0.89	0.593
Estimated glomerular filtration rate (mL/min/1.73 m^2^)	70.19 ± 19.66	78.39 ± 17.85	<0.001
Iron (μg/L)	1192.78 (895.34,1517.69)	1209.31 (928.19,1519.08)	0.366
Copper (μg/L)	856.50 (674.58,1174.97)	855.12 (682.26,1110.23)	0.254
Zinc (μg/L)	1302.97 (1055.19,2666.08)	1511.75 (1158.21,2435.45)	<0.001
Selenium (μg/L)	67.34 (56.33,82.30)	71.65 (59.87,85.26)	<0.001
Manganese (μg/L)	3.28 (2.37,4.82)	2.95 (2.13,4.45)	<0.001
Molybdenum (μg/L)	1.62 (1.22,2.18)	1.41 (1.10,1.85)	<0.001
Vanadium (μg/L)	3.19 (1.68,4.14)	2.86 (1.13,4.09)	<0.001
Cobalt (μg/L)	0.25 (0.19,0.32)	0.24 (0.17,0.31)	<0.001
Chromium (μg/L)	5.41 (3.06,7.91)	5.07 (3.12,7.56)	0.289
Nickel (μg/L)	3.32 (2.08,5.12)	3.09 (1.91,4.55)	0.001
Tin (μg/L)	0.16 (0.07,0.29)	0.16 (0.07,0.28)	0.031

**Table 2:** Hazard ratios (95%CIs) for all-cause and cardiovascular mortality in patients with type 2 diabetes.

Metals (μg/L)	Hazard Ratio (95%CIs) by Continuous Metals	*p*	Hazard Ratio (95%CIs) by Quartiles of Metals				*p* Trend *
			Quartile1	Quartile2	Quartile3	Quartile4	
All-cause mortality ^#^							
Iron	0.83 (0.70,0.98)	0.031	1.00 (ref.)	0.91 (0.75,1.09)	0.81 (0.67,0.98)	0.79 (0.64,0.96)	0.006
Copper	1.60 (1.30,1.97)	<0.001	1.00 (ref.)	1.06 (0.87,1.28)	1.05 (0.86,1.28)	1.50 (1.23,1.82)	<0.001
Zinc	0.96 (0.89,1.03)	0.289	1.00 (ref.)	0.80 (0.66,0.96)	0.96 (0.78,1.17)	0.94 (0.79,1.13)	0.963
Selenium	0.60 (0.46,0.77)	<0.001	1.00 (ref.)	0.82 (0.68,0.98)	0.72 (0.59,0.88)	0.72 (0.58,0.88)	<0.001
Molybdenum	1.08 (0.97,1.20)	0.141	1.00 (ref.)	0.92 (0.74,1.14)	1.10 (0.90,1.34)	1.17 (0.96,1.43)	0.023
Cardiovascular mortality ^&^							
Iron	0.61 (0.49,0.78)	<0.001	1.00 (ref.)	0.70 (0.51,0.95)	0.69 (0.51,0.93)	0.52 (0.38,0.72)	<0.001

#: Metals were ln-transformed and simultaneously included in the Cox model, with adjustment for age, gender, BMI, education, smoking status, drinking status, physical activity status, baseline FBG, duration of diabetes, use of antidiabetics and eGFR at baseline. ^&^: Metals were ln-transformed and simultaneously included in the Cox model, with adjustment for age, gender, BMI, education, smoking status, drinking status, physical activity status, baseline FBG, duration of diabetes and use of antidiabetics, family history of CVD, and eGFR at baseline. *: *p* trend across quartile of metals were obtained by including the median of each quartile (natural ln-transformed) as a continuous variable in the Cox models.

**Table 3:** Hazard ratios (95%CIs) for all-cause and cardiovascular mortality in patients with type 2 diabetes according to the combined categories of plasma metal levels.

Metals ^#^	*n* (Death/Survivors)	Hazard Ratio (95%CIs) ^&^	*P*_interaction_
Iron-Selenium			
Low Fe + Low Se	277/1242	1.00 (ref)	
Low Fe + High Se	179/941	0.94 (0.77,1.13)	0.285
High Fe + Low Se	227/893	0.89 (0.74,1.06)	
High Fe + High Se	207/1312	0.66 (0.55,0.79)	
			
Iron-Copper			
Low Fe + Low Cu	212/1059	1.00 (ref)	
Low Fe + High Cu	244/1124	1.21 (1.00,1.46)	
High Fe + Low Cu	232/1136	0.83 (0.69,1.00)	0.575
High Fe + High Cu	202/1069	0.90 (0.74,1.09)	
			
Selenium-Copper			
Low Se + Low Cu	286/1272	1.00 (ref)	
Low Se + High Cu	218/863	1.17 (0.98,1.40)	
High Se + Low Cu	158/923	0.77 (0.63,0.94)	0.663
High Se + High Cu	228/1330	0.94 (0.79,1.13)	

#: Low is defined as “Q1 + Q2”, High is defined as “Q3 + Q4”. ^&^: Adjusted for age, gender, BMI, education, smoking status, drinking status, physical activity status, baseline FBG, duration of diabetes, and use of antidiabetics and eGFR at baseline.

## Data Availability

The original contributions presented in the study are included in the article/Supplementary Materials, further inquiries can be directed to the corresponding authors.

## Supplementary Materials

The following supporting information can be downloaded at: https://www.mdpi.com/article/10.3390/nu15051198/s1, Figure S1. Flowchart of the participants included in the present study. Figure S2. Spearman correlation heatmap of plasma metals. Figure S3. Adjusted hazard ratios (95%CI) for all-cause mortality among type 2 diabetes individuals with higher level of iron. Figure S4. Adjusted hazard ratios (95%CI) for all-cause mortality among type 2 diabetes individuals with higher level of copper. Figure S5. Adjusted hazard ratios (95%CI) for all-cause mortality among type 2 diabetes individuals with higher level of selenium. Figure S6. Adjusted hazard ratios (95%CI) for CVD mortality among type 2 diabetes individuals with higher level of iron. Figure S7. The restricted cubic spline for the association between plasma iron and CVD mortality among type 2 diabetes. Table S1. Limits of detection and percentages of samples below detection limits of study participants at baseline (*n* = 5278). Table S2. The coefficient profiles for the metals derived from the LASSO regression model. Table S3. Sensitivity analyses to explore the hazard ratios (95%CI) for all-cause and cardiovascular mortality in patients with type 2 diabetes.
