# Supplementary material for: Associations between Plasma Essential Metals Levels and the Risks of All-Cause Mortality and Cardiovascular Disease Mortality among Individuals with Type 2 Diabetes
Source: Nutrients. 2023 Feb 27;15(5):1198. doi: 10.3390/nu15051198 (PMC10005369; doi:10.3390/nu15051198)
Supplement: Supplementary file 1 [file nutrients-15-01198-s001.zip › nutrients-2188872-supplementary.pdf]

Table of Contents

Figure S1. Flowchart of the participants included in the present study.

Figure S2. Spearman correlation heatmap of plasma metals.

Figure S3. Adjusted hazard ratios (95%CI) for all-cause mortality among type 2 diabetes individuals with higher level of iron.

Figure S4. Adjusted hazard ratios (95%CI) for all-cause mortality among type 2 diabetes individuals with higher level of copper.

Figure S5. Adjusted hazard ratios (95%CI) for all-cause mortality among type 2 diabetes individuals with higher level of selenium.

Figure S6. Adjusted hazard ratios (95%CI) for CVD mortality among type 2 diabetes individuals with higher level of iron.

Figure S7. The restricted cubic spline for the association between plasma iron and CVD mortality among type 2 diabetes.

Table S1. Limits of detection and percentages of samples below detection limits of study participants at baseline (n = 5278).

Table S2. The coefficient profiles for the metals derived from the LASSO regression model

Table S3. Sensitivity analyses to explore the hazard ratios (95%CI) for all-cause and cardiovascular mortality in patients with type 2 diabetes.

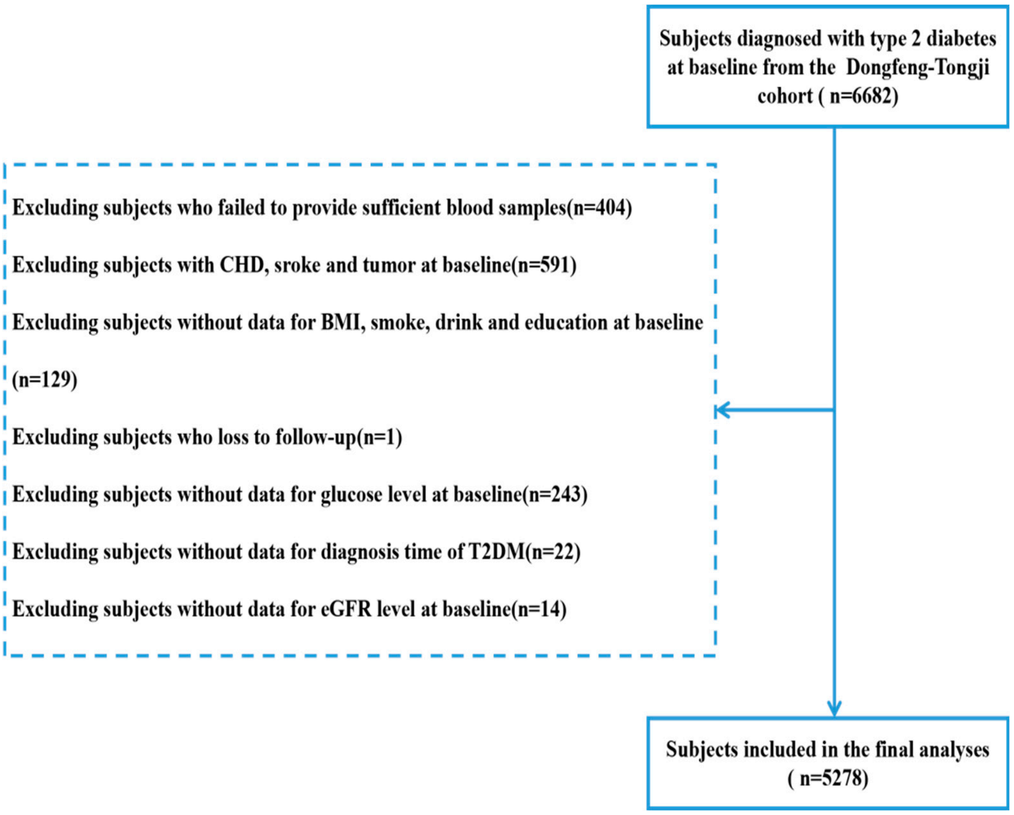

Figure S1. Flowchart of the participants included in the present study.

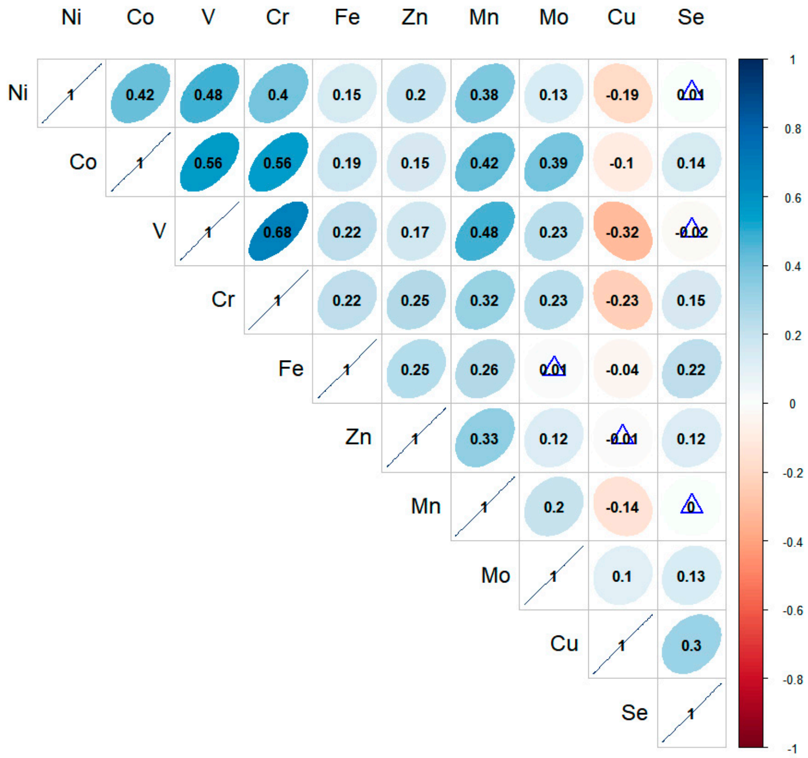

Figure S2. Spearman correlation heatmap of plasma metals. Metals without significant correlations are marked with blue triangles.

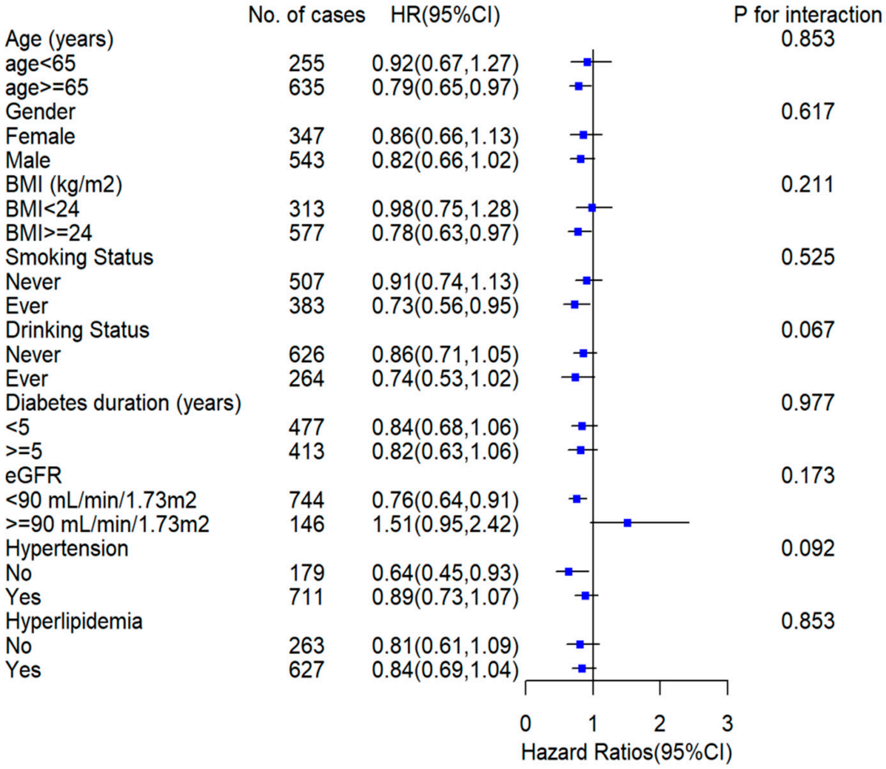

Figure S3. Adjusted hazard ratios (95% CI) for all-cause mortality among type 2 diabetes individuals with higher level of iron. All covariates were age, sex, BMI, education, smoking status, drinking status, physical activity status, baseline FBG, duration of diabetes, use of antidiabetics and eGFR at baseline. Each group adjusted for the other covariates except itself.

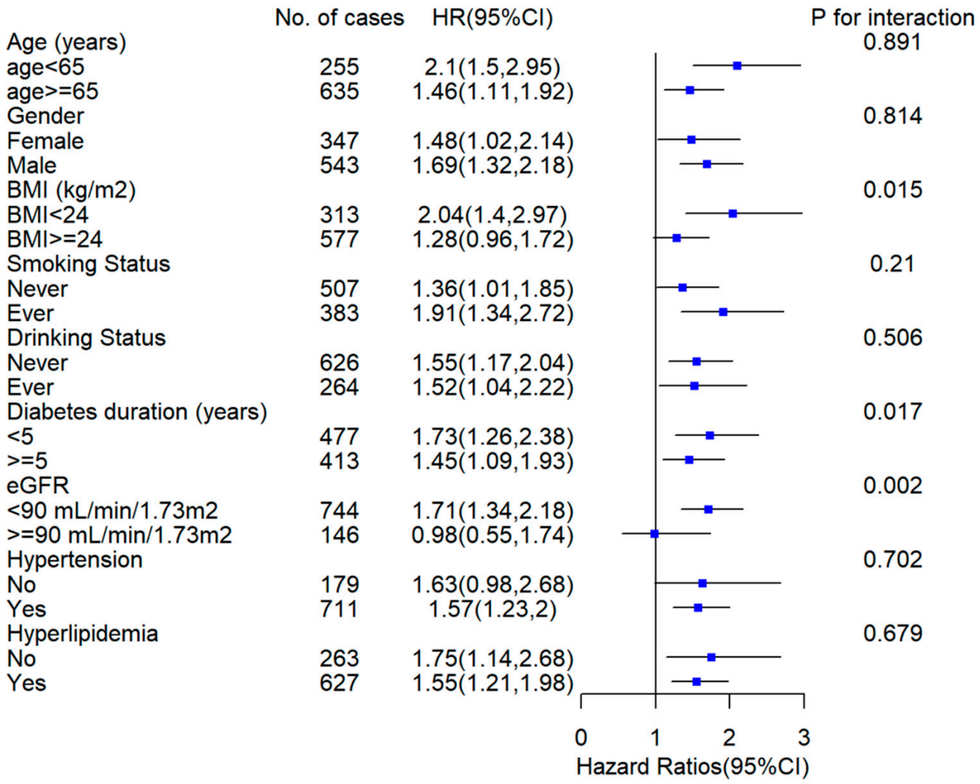

Figure S4. Adjusted hazard ratios (95% CI) for all-cause mortality among type 2 diabetes individuals with higher level of copper. All covariates were age, sex, BMI, education, smoking status, drinking status, physical activity status, baseline FBG, duration of diabetes, use of antidiabetics and eGFR at baseline. Each group adjusted for the other covariates except itself.

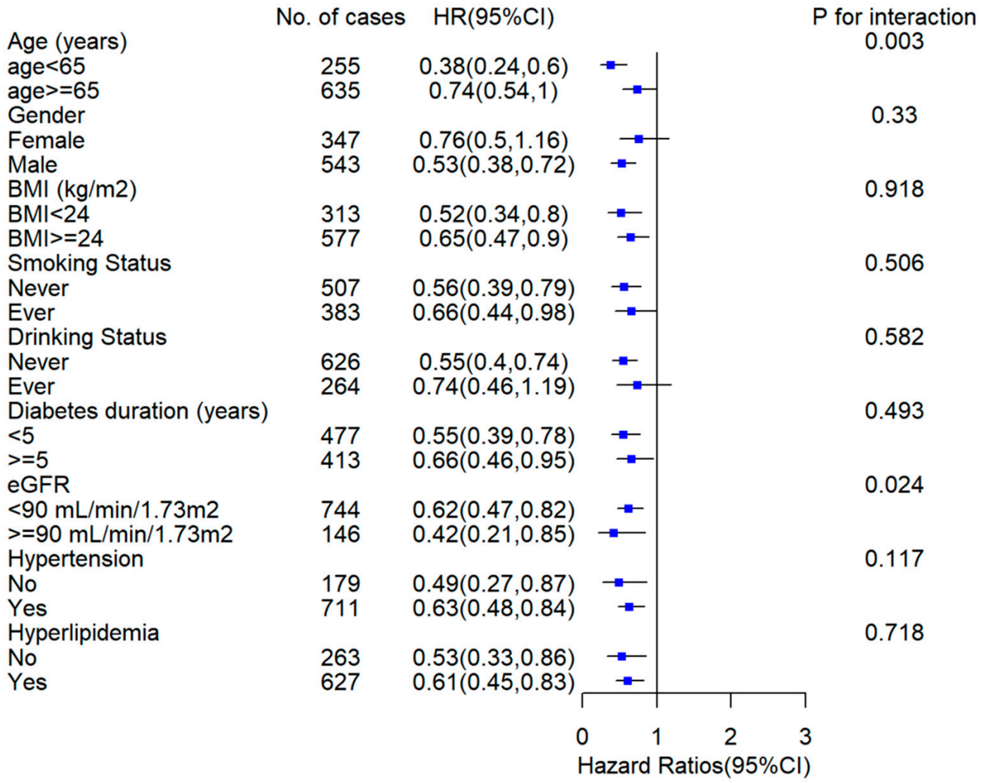

Figure S5. Adjusted hazard ratios (95% CI) for all-cause mortality among type 2 diabetes individuals with higher level of selenium. All covariates were age, sex, BMI, education, smoking status, drinking status, physical activity status, baseline FBG, duration of diabetes, use of antidiabetics and eGFR at baseline. Each group adjusted for the other covariates except itself.

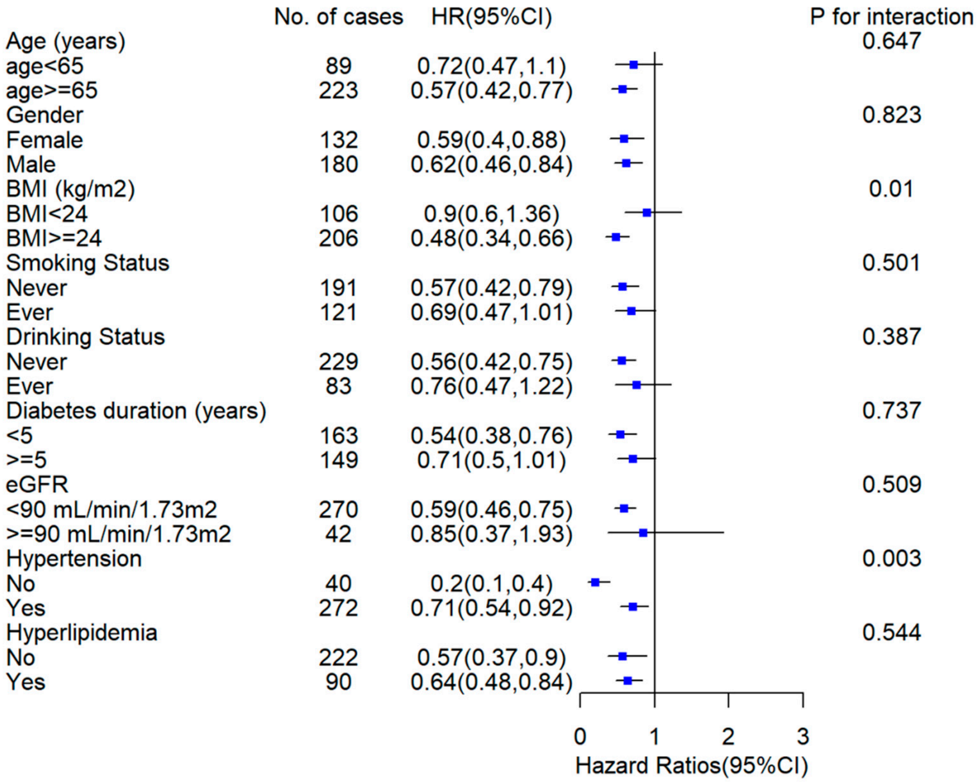

Figure S6. Adjusted hazard ratios (95% CI) for CVD mortality among type 2 diabetes individuals with higher level of iron. All covariates were age, sex, BMI, education, smoking status, drinking status, physical activity status, family history of CVD, baseline FBG, duration of diabetes, use of antidiabetics and eGFR at baseline. Each group adjusted for the other covariates except itself.

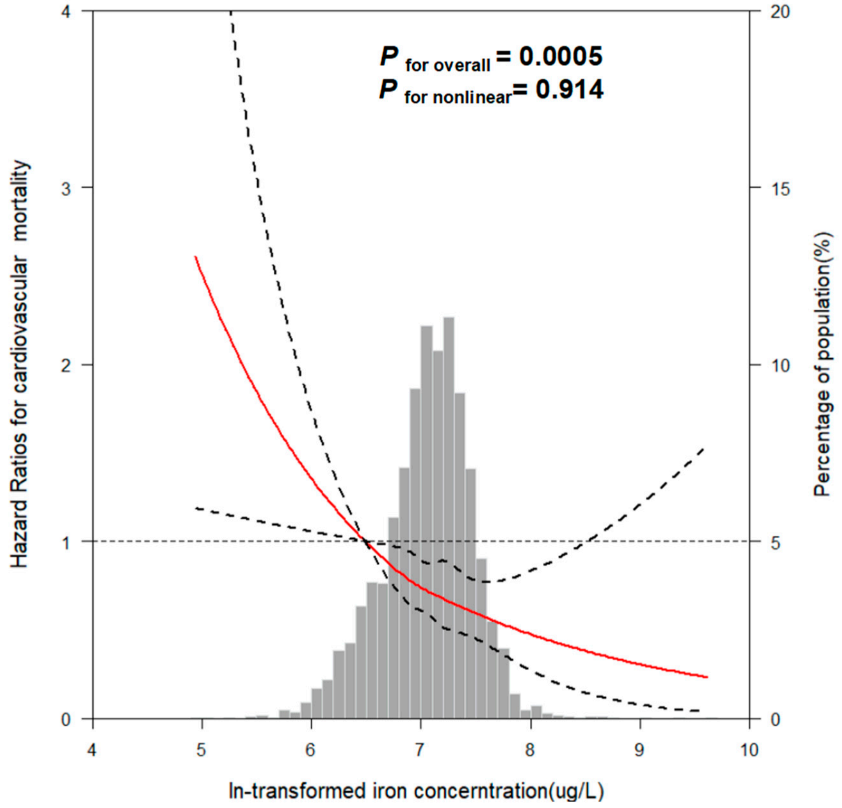

Figure S7. The restricted cubic spline for the association between plasma iron and CVD mortality among type 2 diabetes. Knots were placed at the 20th, 40th, 60th and 80th percentile of the distribution of plasma metal concentration, with the reference value were set at the 10th percentile. The black line was hazard ratio; dashed lines were 95% confidence intervals. Adjustment factors were age, sex, BMI, education, smoking status, drinking status, physical activity status, family history of CVD, baseline FBG, duration of diabetes, use of antidiabetics and eGFR at baseline.

Table S1. Limits of detection and percentages of samples below detection limits of study participants at baseline (n = 5278).

Plasma metals (μg/L)	LOD	Total No. (%) < LOD
Iron	0.17637	0(0.00)
Copper	0.00384	1(0.02)
Zinc	0.21972	0(0.00)
Selenium	0.00969	1(0.02)
Manganese	0.00507	24(0.46)
Molybdenum	0.00126	44(0.83)
Vanadium	0.00129	0(0.00)
Cobalt	0.00099	89(1.69)
Chromium	0.0204	21(0.39)
Nickel	0.01146	65(1.23)
Tin	0.003	1008(19.09)

Abbreviations: LOD, limit of detection.

Table S2. The coefficient profiles for the metals derived from the LASSO regression model.

Metals	Coefficients	
	All-cause mortality[#]	Cardiovascular mortality[&]
Iron	-0.123	-0.306
Copper	0.358	/
Zinc	-0.020	/
Selenium	-0.399	/
Chromium	/	/
Manganese	/	/
Molybdenum	0.023	/
Cobalt	/	/
Nickel	/	/
Tin	/	/
Vanadium	/	/

#: Metals were ln-transformed and simultaneously included in the LASSO model, with adjustment for age, gender, BMI, education, smoking status, drinking status, physical activity status,baseline FBG, duration of diabetes, use of antidiabetics and eGFR at baseline. &: Metals were ln-transformed and simultaneously included in the Cox model, with adjustment for age, gender, BMI, education, smoking status, drinking status, physical activity status, baseline FBG, duration of diabetes and use of antidiabetics, family history of CVD, and eGFR at baseline.

Table S3. Sensitivity analyses to explore the hazard ratios (95% CI) for all-cause and cardiovascular mortality in patients with type 2 diabetes.

Metals (μg/L)	Hazard ratio (95% CIs) by continuous metals	*P*	Hazard ratio (95% CIs) by quartiles of metals				P_{trend}*
			Quartile1	Quartile2	Quartile3	Quartile4	
All-cause mortality[#]							
Excluding the subjects who died within the first 2 years of follow-up (n=106)							
Iron	0.86(0.72,1.03)	0.095	1.00 (ref.)	0.91(0.75,1.12)	0.83(0.68,1.03)	0.81(0.65,1.00)	0.022
Copper	1.50(1.21,1.87)	<0.001	1.00 (ref.)	1.11(0.91,1.35)	1.13(0.91,1.40)	1.42(1.15,1.76)	0.004
Zinc	0.97(0.90,1.05)	0.46	1.00 (ref.)	0.77(0.63,0.94)	0.94(0.75,1.17)	0.96(0.80,1.16)	0.631
Selenium	0.62(0.48,0.81)	0.001	1.00 (ref.)	0.81(0.67,0.99)	0.72(0.58,0.88)	0.77(0.62,0.96)	0.002
Molybdenum	1.11(0.99,1.25)	0.075	1.00 (ref.)	0.88(0.70,1.11)	1.13(0.91,1.40)	1.19(0.96,1.48)	0.018
Excluding the subjects whose eGFR<60mL/min/1.73 m^2 at baseline (n=868)							
Iron	0.87(0.71,1.07)	0.178	1.00 (ref.)	0.85(0.68,1.06)	0.78(0.62,0.99)	0.79(0.62,1.00)	0.022
Copper	1.62(1.29,2.03)	<0.001	1.00 (ref.)	1.08(0.86,1.34)	0.97(0.77,1.23)	1.36(1.07,1.73)	0.062
Zinc	0.98(0.90,1.07)	0.661	1.00 (ref.)	0.79(0.64,0.99)	0.91(0.72,1.16)	0.94(0.76,1.17)	0.856
Selenium	0.55(0.41,0.74)	<0.001	1.00 (ref.)	0.77(0.62,0.95)	0.65(0.51,0.82)	0.66(0.52,0.85)	<0.001
Molybdenum	1.00(0.91,1.11)	0.925	1.00 (ref.)	0.90(0.71,1.15)	1.02(0.81,1.28)	1.09(0.86,1.37)	0.23
Excluding the subjects who died accidentally during follow-up (n=21)							
Iron	0.83(0.70,0.99)	0.035	1.00 (ref.)	0.92(0.76,1.11)	0.81(0.67,0.99)	0.79(0.64,0.97)	0.007
Copper	1.62(1.31,2.01)	<0.001	1.00 (ref.)	1.05(0.87,1.28)	1.04(0.85,1.27)	1.50(1.23,1.84)	<0.001
Zinc	0.96(0.89,1.03)	0.271	1.00 (ref.)	0.79(0.66,0.96)	0.94(0.77,1.16)	0.93(0.78,1.12)	0.95
Selenium	0.61(0.47,0.78)	<0.001	1.00 (ref.)	0.83(0.69,1.00)	0.73(0.60,0.89)	0.74(0.6,00.91)	0.001

Molybdenum	1.08(0.97,1.20)	0.146	1.00 (ref.)	0.93(0.75,1.15)	1.12(0.91,1.37)	1.18(0.96,1.45)	0.02
Cardiovascular mortality[&]							
Excluding the subjects who died of CVD within the first 2 years of follow-up (n=20)							
Iron	0.61(0.48,0.78)	<0.001	1.00 (ref.)	0.68(0.49,0.94)	0.71(0.52,0.96)	0.53(0.38,0.74)	<0.001
Excluding the subjects whose eGFR<60mL/min/1.73 m^2 at baseline (n=868)							
Iron	0.62(0.47,0.81)	0.001	1.00 (ref.)	0.68(0.47,0.98)	0.58(0.40,0.84)	0.53(0.36,0.77)	<0.001
Excluding the subjects who died accidentally during follow-up (n=21)							
Iron	0.61(0.49,0.78)	<0.001	1.00 (ref.)	0.7(0.51,0.95)	0.68(0.5,0.93)	0.52(0.37,0.71)	<0.001

#: Metals were ln-transformed and simultaneously included in the Cox model, with adjustment for age, sex, BMI, education, smoking status, drinking status, physical activity status, baseline FBG, duration of diabetes, use of antidiabetics and eGFR at baseline.

&: Metals were ln-transformed and simultaneously included in the Cox model, with adjustment for age, sex, BMI, education, smoking status, drinking status, physical activity status, baseline FBG, duration of diabetes and use of antidiabetics, family history of CVD, and eGFR at baseline.

*: *P* trend across quartile of metals were obtained by including the median of each quartile (natural ln-transformed) as a continuous variable in the Cox models.
